# HCV Co-Infection and Its Genotypic Distribution in HIV-Infected Patients in Nepalese Population

**DOI:** 10.3390/tropicalmed8070361

**Published:** 2023-07-13

**Authors:** Uday Kant Sah, Anil Kumar Sah, Mehraj Ansari, Priyanka Chaudhary, Saurav Gupta, Pawan Kumar, Jay Prakash Sah

**Affiliations:** 1 Department of Biochemistry, Singhania University, Pacheri Bari, Rajasthan 333515, India; 2 Annapurna Research Center, Kathmandu 44600, Nepal; 3 Shi-Gan International College of Science and Technology, Kathmandu 44600, Nepal; 4 Patan Academy of Health Sciences, Lalitpur 44700, Nepal; 5 Department of Neuroscience, The University of Alabama at Birmingham, Birmingham, AL 35294, USA; 6 School of Health and Allied Sciences, Pokhara University, Pokhara-30, Kaski 33700, Nepal; 7 Division of Molecular and Cellular Pathology, Department of Pathology, The University of Alabama at Birmingham, Birmingham, AL 35233, USA

**Keywords:** HCV genotypes, HCV viral load, HIV, PLHIV

## Abstract

Hepatitis C Virus (HCV) co-infection and its genotypic distribution in people living with Human Immunodeficiency Virus (HIV) show global inconsistency. Therefore, the present study aimed to investigate the prevalence and genotypic distribution patterns of HCV, along with viral load, in people living with HIV. This cross-sectional study was conducted at SRL Diagnostics Nepal, Pvt. Ltd. in 203 HIV-seropositive patients attending the Tribhuvan University Teaching Hospital (TUTH), Maharajgunj, Kathmandu, Nepal from October 2021 to May 2022. The viral load and HCV genotypes were estimated from RNA extracted from the blood sample (plasma) of PLHIV by using a standard Q-PCR protocol. HCV infection was considered as a core variable, whereas covariates used for this study were duration of HIV infection, age, sex, and ART regimen. Out of total 203 PLHIV, the estimated prevalence of HCV co-infection was 115 (56.6%). Male gender was a unique characteristic associated with a high prevalence of HCV co-infection compared to females. The HCV viral load among PLHIV ranged from 34 to 3,000,000 IU/mL. Among HCV co-infected PLHIV, 56 (48.69%) had a low level of HCV viral load. Interestingly, only 3 (2.6%) patients had an HCV viral load higher than 3,000,000 IU/mL. Diverse HCV genotypes were found in the population, including genotypes 1, 1a, 3a, 5a, and 6. However, genotype 3 was the most prevalent HCV variant among HCV-co-infected PLHIV, with a distribution of 36 (61.1%) and viral load ranging from 34 to 3000 IU/mL. HCV co-infection is frequent in the Nepalese population of people living with HIV, particularly due to HCV genotypic variant 3. The findings of this study could be useful for the management and clearance of the HCV co-infection in PLHIV, aiming to provide a good quality of life.

## 1. Introduction

Hepatitis C Virus (HCV) infection is a major global health problem, significantly impacting the quality of life, morbidity, mortality, and the social and economic development of people living with HIV (PLHIV) [1]. Pathogenically, HCV leads to life-threatening liver complication along with many other abnormalities, including immune activation and various organs’ chronic inflammation. These abnormalities ultimately account for an increased risk of cardiovascular events, kidney disease, and cancers [2]. Even though the treatment of HCV infection has undergone a revolution with simplified treatment regimens with increased tolerability and efficacy, treatment still faces many challenges for the remedy of this infection due to the significant influence of HCV genotypes and their viral load to therapy [3,4]. Mavyret (Glecaprevir, an HCV NS3/4A protease inhibitor/Pibrentasvir, an HCV NS5A inhibitor) is a potent drug for patients with HCV genotypes 2–6 without cirrhosis or with mild cirrhosis [5], whereas Zepatier and Grazoprevir are highly effective for HCV genotypes including 1a, 1b and 4 [6]. Therefore, treatment regimens based on genotypic pattern of HCV and their viral load are required.

Globally, it is estimated that 36.7 million people are infected with human immunodeficiency virus (HIV), and 6.3% of them are co-infected with hepatitis C virus (HCV) [2]. The prevalence of HCV genotypes and subtypes vary significantly across different parts of the world. Generally, genotypes 1 and 3 are the most common genotypes worldwide [7,8]. Approximately 46% of HCV cases are related to genotype 1, while genotype 3 accounts for around 30%. [8]. However, certain genotypes are more dominant in specific geographic areas. In Central and Northern Europe, as well as the United States, genotype 1 (subtypes 1a and 1b) is more prevalent, while HCV subtypes 2a, 2b, 3a, 4a, and others occur less frequently [8,9,10]. HCV genotype 2, along with its subtypes, is also known to be the most dominant genotype in Asia and is also commonly found in the Mediterranean countries and Northern Europe. HCV subtype 4a is predominant in the Middle East and North/Central Africa [11,12,13]. Furthermore, genotypes 5 and 6 are mainly confined to South Africa and South-East Asia, respectively [12,13].

In Nepal, only a few studies have reported the prevalence of HIV and HCV with genotypes [14,15]. Therefore, regular monitoring of HCV genotypic patterns and their viral loads is a basic need to provide effective treatment for HCV co-infected PLHIV. Hence, the present study aims to determine the prevalence of HCV co-infection and its genotypic patterns along with their viral loads in the Nepalese population.

## 2. Materials and Methods

### 2.1. Study Design and Sample Collection

This cross-sectional study was conducted from October 2021 to May 2022 in HIV-seropositive patients attending the Tribhuvan University Teaching Hospital (TUTH), Kathmandu, Nepal. The diagnosed PLHIVs were further confirmed to be HIV-seropositive under the national HIV testing algorithm of the National Centre for AIDS and STD Control (NCASC). The patients were randomly selected and were of all ages, sexes, and social classes with different ART status. Information regarding sex, date of HIV diagnosis, ART duration, and type of ART was obtained before sample collection. In addition, clinically relevant information was also obtained from the logbook of the hospital. 

Venous blood samples were aseptically collected from the participants as described previously [16], and the plasma was screened for anti-HIV antibodies using Retroquic HIV test kit (Qualpro Diagnostics, Verna, Goa, India). Remaining plasma samples were stored at −80 °C until tested for HCV. All procedures were conducted under the aseptic conditions in BSL-2 laboratory, following WHO guidelines.

### 2.2. Inclusion and Exclusion Criteria 

The study included patients of all ages, while individuals who did not wish to participate were excluded.

### 2.3. Screening of HCV Co-Infection

The screening of HCV co-infection was performed with an HCV rapid test kit (Bioline Diagnostics LLP, Delhi, India) based on double-antigen lateral flow chromatographic immunoassay to quantitively determine anti-hepatitis C virus antibodies (IgG, IgM, IgA) in human serum or plasma.

### 2.4. Viral Nucleic Acid Extraction

HCV RNA was extracted using a QIAamp DSP virus kit (Qiagen, Hilden, Germany), following the manufacturer’s instructions with slight modifications. Briefly, 200 μL of plasma was mixed with 25 μL protease reagent and 200 μL Buffer AL then incubated at 56 °C for 15 min. The lysates were mixed in 250 μL of ethanol (96–100%), incubated at room temperature for 5 min, and transferred to the QIAamp DSP Virus column for centrifugation at 6000× *g* for 1 min. Next, 500 μL of Buffer AW1/AW2 was added into the column and centrifuged at 6000× *g* for 1 min. The bottom part of column tube was replaced with a sterile collection tube, which was then centrifuged at 20,000× *g* for 3 min and dried at 56 °C for 3 min on a heating block. The clean virus RNA sample was eluted in 60 μL of Buffer AVE after centrifugation at 20,000× *g* for 1 min. The extracted RNAs were stored at −80 °C for further analysis.

### 2.5. Quantitative RT-PCR

Quantitative RT-PCR was performed using Artus HCV QS-RGQ kit (Qiagen, Hilden, Germany) and QuantStudio 5 machine (Thermo Fisher Scientific, Waltham, MA, USA) to estimate the viral load of HCV RNA. Briefly, the viral RNA was reverse transcribed into complementary DNA using RT-master mixture according to the manufacturer’s protocol. Quantitative PCR was then conducted using the cDNA. The quantitative PCR condition included incubation at 95 °C for 15 min, followed by 50 cycles of amplification with denaturation at 95 °C for 30 s, annealing at 50 °C for 1 min, and extension at 72 °C for 30 s. The HCV viral load in IU/mL was determined using the calculation provided in the protocol.

### 2.6. HCV Genotyping

HCV RNA positive samples with viral load > 34 IU/mL were analyzed for HCV genotypes (1a, 1, 2 (2a/2b), 3, 4, 5a and 6) using TRUPCR^®^ HCV genotyping kit (Cat- 3B253, Bhopal, India). Briefly, a 7μL reaction mixture (6μL of RT-mix and 1μL of enzyme mix) was mixed with 10μL of extracted RNA sample and incubated at 42 °C for 60 min, followed by 5 min of incubation at 95 °C for complementary DNA synthesis. Three different PCR conditions were used for HCV genotyping: condition 1 for genotype 1 and 5a, condition 2 for genotypes 1a, 4 and IC, and condition 3 for genotypes 2 (2a/2b), 3, and 6. The PCR reaction conditions included incubation at 94 °C for 10 min for a single cycle, followed by 40 cycles of incubation with denaturation at 94 °C for 15 s, annealing at 58 °C for 45 s, and extension of primer at 72 °C for 15 s. The cut off value for this assay was 37 cycles. 

### 2.7. Quality Control

The entire tests were performed using ISO- and CE-IVD-certified reagents and chemicals under highly aseptic conditions, following standard operative procedures (SOPs). Positive and negative controls were used in each batch/step. The reagents and samples were stored and incubated at verified temperatures. The inclusion and exclusion criteria were carefully monitored.

### 2.8. Data Management and Analysis

All collected data were entered into the MS Excel sheet, and statistical analyses were conducted using SPSS v20.0. Fisher’s exact and *χ*^2^ tests were performed to determine the significance of differences among categorical variables, and *t*-tests were used for continuous variables. The data were summarized and presented in a descriptive measure such as a table, figures, and line graphs. Statistical tests were two-tailed, and *p*-value < 0.05 confirmed statistically significance. 

## 3. Results

### 3.1. Prevalence of HCV Co-Infection in the People Living with HIV (PLHIV)

The study enrolled a total of 203 people living with HIV (PLHIV), who were confirmed under the national HIV testing algorithm of the national center for AIDS and STD control. Out of 203 participants, male and female were 148 (72.9%) and 55 (27.1%) respectively (Table 1). The age of PLHIV in this study ranged from 5 years to 70 years.

To determine the prevalence of HCV-co-infection among PLHIV, we conducted qRT-PCR from the plasma of patients to detect HCV RNA. Out of 203,115 (56.65%) patients were found to be HCV co-infected (Table 1). Of these, most of the HCV-co-infected PLHIV were male (110; 95.6%), while only a small number were female (5; 4.4%) (Table 2). The mean age for the HCV-co-infected PLHIV was 43.45 ± 0.594 years (Table 3). Although it was not statistically significant (*p* = 0.21), females were relatively younger (40.0 ± 0.56 years) compared to males (43.6 ± 3.62 years) (Table 3). 

The highest prevalence of PLHIV co-infected with HCV were observed in the age group of 25–50 years (Table 2). Interestingly, the young PLHIVs (<15 years) were 17 (8.37%) and were not co-infected with HCV (Table 2), suggesting the possibility that young people may have a strong immune response that can resist HCV infection compared to adults. Furthermore, HCV co-infection was also not observed in youth PLHIV (15–24 years), even though their population in this study was good enough (42, 20.7%) (Table 1).

### 3.2. Prevalence of HCV Genotype in PLHIV in Nepalese Population

Out of 115 HCV-co-infected PLHIV, the majority of patients (56; 48.69%) had a low level of HCV viral load (Table 4). Specifically, 56 (48.69%) patients had a poor range of HCV viral load, which was less than 34 IU/mL. In contrast, 31 (26.9%) HCV-co-infected PLHIV had viral load in the range of 34–3000 IU/mL. The least number of PLHIV had very high levels of HCV viral load, which exceeded 3,000,000 IU/mL (Table 4). Surprisingly, Genotype 3 was found to be the most prominent HCV variant in HCV co-infected PLHIV in this study; its distribution was 36 (61.1%). On the other hand, genotypes 5a, 6, 1, and 1a were poorly distributed in the Nepalese population. The distribution patterns of genotypes 5a, 6, 1, and 1a were 13 (22.1%), 6 (10.2%, 2 (3.3%), and 2 (3.3%), respectively (Table 4). 

Next, we checked the correlation between viral load and the HCV genotyping. The significant association between viral load and HCV genotyping was found (*p* = 0.00) (Table 4), propounding the fact that HCV infection, pathogenesis and its therapeutic aspects may be based on its genotype. Several studies also explore that HCV treatment should be tailored based on HCV genotype and viral load [5,6,17]. 

We also checked the correlation between age and viral load. PLHIV in the age group of 25–50 years had high viral loads, while older PLHIV (>50 years) had the lowest (Table 4). Additionally, individuals who had been living with HIV for a duration of 15 years had a higher incidence of HCV co-infection, whereas those with less than 5 years of HIV infection had a lower rate of HCV co-infection. There was a significant association between HIV duration and HCV viral load (*p* = 0.010) (Table 4). Taken together, we explore the fact that HCV co-infection in PLHIV may be dependent on the duration of HIV infection, possibly due to the weakened immune system in the people with prolonged HIV infection [18].

Table 5 shows the distribution pattern of HCV genotype by sex and age in PLHIV. Genotype 3 was the most dominant HCV variant among both males—34 (59.6%)—and females—2 (100%)—in the Nepalese population. However, the association between HCV genotype distribution and sex was not statistically significant, possibly due to the low sample size of females (*p* = 0.858). When we checked the distribution pattern of HCV genotypes by age, genotype 3 was found to be prominent across all age groups in PLHIV (Table 5). Consistent with the above finding, we also found genotype 3 as the most dominant HCV variant in all groups of HIV/ART duration (Table 5). These correlations were not statistically significant which might be due to the small sample size. Overall, these findings reveal that genotype 3 is the most prominent HCV variant among HCV-co-infected PLHIV in the Nepalese population.

## 4. Discussion

HCV co-infection is one of the common leading causes of liver-related death in people living with HIV/AIDS. Several studies report that HCV shares similar route of transmission with HIV and leads to increase the frequency and severity of liver disease, which ultimately increases the morbidity and mortality rate of PLHIV [19,20,21,22]. However, in developing countries, the management of HCV co-infection has received comparatively less attention compared to HIV infection [23,24]. Therefore, HCV infection and its pathogenic complication should be addressed during the care of co-infected PLHIV to ensure effective treatment and improve their quality of life. 

The overall prevalence of HCV co-infection in this study was 56.6%. In several earlier studies, the prevalence of HCV co-infection in different locations has been estimated at low rates in South India (3.52%) [25], Singapore and Hong Kong (0.5%), Vietnam and Thailand (6%), and Myanmar (10%) [26]. Compared to Kyrgyzstan (49.7%), Spain (33%), the USA (30%), Tajikistan and Uzbekistan (26.5%), France (24.3%), Morocco (19.8%), and a previous study from Nepal (43.3%), the present prevalence was still high [27,28,29,30]. Overall, our figure is higher than findings from many other Asian countries and other continents. These variations could be attributed to differences in sociodemographic characteristics and the immune status of PLHIV. The other possibility in some cases may be due to loss of hope to survive and to putting these people back into the previous life through which they became infected.

In this study, the majority of HCV co-infected PLHIV were male (110; 95.6%) compared to females (5; 4.4%) (Table 2). This finding was not statistically significant, possibly due to the higher number of males included in the sample population, and hence, does not justify saying that the gender has a direct influence on HCV co-infection. 

The distribution pattern of HCV-co-infection among PLHIV showed a higher prevalence in the age group of 25–50 years (Table 2). Even though there was no direct association of HCV prevalence with age statistically, our finding is still in agreement with a previous study from Nepal that reported an increased prevalence of HCV co-infection in the age group of 30–39 years [23]. 

HCV co-infection was significantly associated with the duration of HIV infection (Table 4). This finding reflects the fact that HCV may infect PLHIV through opportunistic infection due to their decreased immunity, especially low CD4 counts [18].

Genotype 3 was the most prevalent HCV variant among HCV co-infected PLHIV in the Nepalese population, accounting for 61.1% of cases, while other genotypes such as 5a, 6, 1a, and 1 were poorly distributed (Table 4). The majority of the HCV-co-infected PLHIV with genotypes 3 were in the age group of 25–50 years (Table 4). There was no gender bias in the distribution of genotype 3 among HCV-co-infected PLHIV (Table 4). Consistent with our findings, some earlier studies also report genotypes 3 as a key variant responsible for most HCV infections [23,24]. Likewise, HCV genotypes (1, 1a, 5a, and 6) have also been reported in earlier studies from Asian countries including Nepal [31,32,33]. Moreover, the distribution of genotypes did not show a statistically significant correlation with age, sex, or HIV/ART duration (Table 5). This may be possibly due to the limited sample size in this study. In contrast, a previous study reported a significant association between HCV genotypes and HIV duration [34]. 

The present study showed a significant correlation between HCV genotypes and HIV viral load (Table 4). PLHIV infected with HCV genotype 3 had a significantly lower viral load compared to other genotypes. Even though the number of PLHIV infected with HCV genotype 1a was limited in this study, their viral load was higher compared to all other genotypes. Almost 1/3 part of PLHIV co-infected with HCV did not show a detectible viral load. These observations are well supported by several earlier reports conducted in different study settings [32,33,35,36].

In this study, we found that the prevalence of HCV co-infection in PLHIV was higher in Nepal than in other Asian and non-Asian countries. HCV co-infection was significantly associated with viral load in PLHIV, and genotype 3 was the most prominent HCV co-infection in PLHIV in the Nepalese population. This study exclaims the need for early diagnosis, viral load determination, and genotypic detection of HCV infection in all PLHIV to initiate effective treatment. However, it should be noted that this study had a cross-sectional design and a limited sample size. Further investigations with a large population size are recommended to explore the casual relationships between variables and provide a more comprehensive insight into the clinical, virological, and therapeutic characteristics of HCV co-infection in PLHIV.

## 5. Conclusions

The prevalence of HCV genotype 3 is notably high in the Nepalese population, which makes it the most common genotype among HCV-co-infected PLHIV. Additionally, the viral load for HCV in PLHIV is also at an alarming level. Therefore, accurate knowledge on the distribution pattern of HCV genotypes and their viral loads is essential for implementing potential therapeutic approaches to effectively cope hepatitis C co-infection in the Nepalese population. 

## Figures and Tables

**Table 1:** Characteristics of PLHIV in Nepalese population.

Parameters	Characteristics	Status
HCV co-infection	Positive	115 (56.6%)
	Negative	88 (43.4%)
Sex	Male	148 (72.9%)
	Female	55 (27.1%)
Age Group (Years)	<15	14 (6.9%)
	15–24	29 (14.3%)
	25–50	138 (68.0%)

**Table 2:** Characteristics of HCV-co-infected PLHIV in Nepalese population.

Parameters	Characteristics	Status
Sex	Male	110 (95.6%)
	Female	05 (4.4%)
Age Group (Years)	<15	00 (0.0%)
	15–24	00 (0.0%)
	25–50	102 (86.1%)
	>50	13 (11.3%)

**Table 3:** Characteristics of HCV co-infected PLHIV in Nepalese population.

Parameters	Mean	SEM	*p*-Value
Age (years)			
Male	40.0	0.56	0.21
Female	43.6	3.62	
Both	43.45	0.594	

**Table 4:** Distribution of HCV genotypes based on their viral load and PLHIV characteristics.

Viral Load (IU/mL)	<34	34–3000	3000–30,000	30,000–3,000,000	>3,000,000	Total	*p*-Value
Number of patients							
	56 (48.69%)	31 (26.9%)	17 (14.7%)	8 (6.95%)	3 (2.6%)	115	
HCV genotypes							
1	0 (0.0%)	1 (50.0%)	0 (0.0%)	1 (50.0%)	0 (0.0%)	2 (3.3%)	0.00
1a	0 (0.0%)	0 (0.0%)	0 (0.0%)	0 (0.0%)	2 (100%)	2 (3.3%)	
3	0 (0.0%)	22 (61.1%)	9 (25.0%)	4 (11.1%)	1 (2.8%)	36 (61.1%	
5a	0 (0.0%)	4 (30.8%)	7 (53.8%)	2 (15.4%)	0 (0.0%)	13 (22.1%)	
6	0 (0.0%)	4 (66.7%)	1 (16.7%)	1 (16.7%)	0 (0.0%)	6 (10.2%)	
Gender							
Male	53 (48.2%)	29 (26.4%)	17 (15.5%)	8 (7.3%)	3 (2.7%)	110 (95.6%)	0.783
Female	3 (60.0%)	2 (40.0%)	0 (0.0%)	0 (0.0%)	0 (0.0%)	5 (4.4%)	
Age Groups (Years)							
25–50	51 (50.0%)	25 (24.5%)	16 (15.7%)	7 (6.9%)	0 (0.0%)	99 (86.1%)	0.523
>50	5 (48.7%)	6 (46.2%)	1 (7.7%)	1 (7.7%)	3 (2.6%)	16 (13.9%)	
HIV/ART Duration (Years)							
1–5	0 (0.0%)	3 (60.0%)	0 (0.0%)	0 (0.0%)	2 (40.0%)	5 (8.5%)	0.010
6–10	0 (0.0%)	20 (58.8%)	8 (23.5%)	5 (14.7%)	1 (2.9%)	34 (57.6%)	
11–15	0 (0.0%)	9 (45.0%)	7 (35.0%)	4 (20.0%)	0 (0.0%)	20 (33.9%)	

**Table 5:** HCV Genotypes distribution based on HIV/ART duration and PLHIV characteristics.

Genotypes	1	1a	3	5a	6	*p* Value
Sex						
Male	2 (3.5%)	2 (3.5%)	34 (59.6%)	13 (22.8%)	6 (10.5%)	0.858
Female	0 (0.0%)	0 (0.0%)	2 (100%)	0 (0.0%)	0 (0.0%)	
Age Groups (years)						
25–50	2 (3.9%)	2 (3.9%)	28 (54.9%	13 (25.5%)	6 (11.8%)	0.206
>50	0 (0.0%)	0 (0.0%)	8 (100.0%)	0 (0.0%)	0 (0.0%)	
HIV/ART Duration (Years)						
1–5	0 (0.0%)	1 (20.0%)	4 (80.0%)	0 (0.0%)	0 (0.0%)	0.341
6–10	1 (2.9%)	1 (2.9%)	18 (52.9%)	10 (29.4%)	4 (11.8%)	
11–15	1 (5.0%)	0 (0.0%)	14 (70.0%)	3 (15.0%)	2 (10.0%)	

## Data Availability

All data supporting the study findings are unavailable due to privacy or ethical restrictions.
